# Arritmia Ventricular Potencialmente Fatal Induzida por Taquicardia Atrial em uma Criança com Mutação de SCN5A

**DOI:** 10.36660/abc.20200509

**Published:** 2021-07-14

**Authors:** Márcio Augusto Silva, Jorge Elias, Guilherme Muller de Campos Futuro, Erick Sessa Merçon, Deborah Vasconcelos, Ricardo Kuniyoshi

**Affiliations:** 1 Vitoria Apart Hospital SerraES Brasil Vitoria Apart Hospital - Cardiologia, Serra, ES - Brasil.

**Keywords:** Arritmias Cardíacas, Síndrome de Brugada, Criança

## Introdução

Mutações no gene SCN5A, que codifica o canal Na+ cardíaco, podem causar arritmias potencialmente fatais. Identificou-se que essas mutações são causadoras de doenças elétricas primárias hereditárias, incluindo a síndrome de Brugada (SB), síndrome do QT longo, e outros distúrbios da condução cardíaca.^1^^,^^2^ A SB, a doença mais relatada desse grupo de transtornos, geralmente é descrita em populações adultas e está relacionada a aproximadamente 20% de todas as mortes súbitas (MS) em pacientes com corações aparentemente normais.^3^^-^^5^ Portanto, alguns relatos mostraram eventos arrítmicos (EA) significativos causados por essa mutação na infância.^6^

Arritmias atriais, bem como a síndrome do nó sinusal (SNS), podem estar relacionadas a anormalidades do canal Na+. Na SB, arritmias atriais são diagnosticadas em até 38% dos pacientes, e estão relacionadas a prognósticos piores.^7^

## Relato de Caso

**Figura 1:**
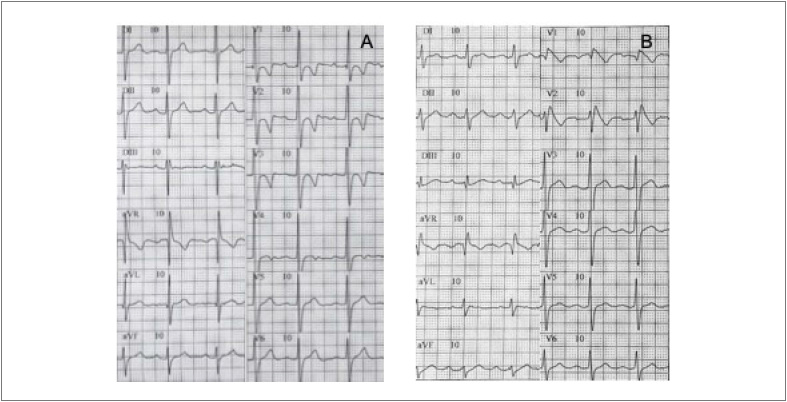
ECG de 12 derivações espontâneas (A) – Criança; (B) – Pai.

Um menino de 2 anos de idade, sem anomalias cardíacas detectadas em exames de ecocardiograma transtorácico e imagens de ressonância magnética (RM), deu entrada duas vezes no hospital com padrão típico de flutter atrial (FLA), que foi revertido por cardioversão elétrica. Após o último episódio, ele recebeu alta com prescrição de 3 mg/kg de amiodarona por dia. O ECG de 12 derivações revelou intervalos QT (390 – 410 ms) e QTc (413 – 440 ms) normais, intervalo PR (200 ms) prolongado, e ondas T negativas nas derivações precordiais direitas (Figura 1).

**Figura 2:**
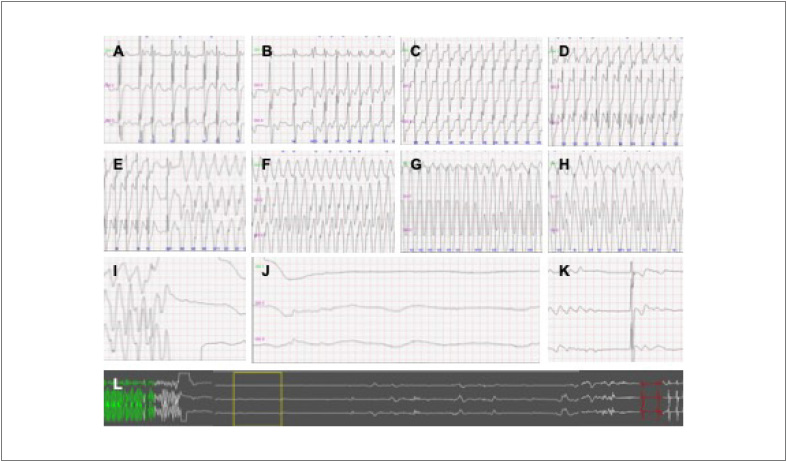
Evento sincopal registrado no Holter de 24 horas. A, B – TA/AFL de condução AV variável, com QRS estreito; C, D – A condução AV passou a 1:1 com alargamento progressivo de QRS; E, F, G, H – TF e TV polimórfica ampla sustentada; I, J, K – a FV é seguida de 30 segundos de assistolia, com recuperação espontânea do ritmo sinusal; L – faixa longa de assistolia total.

Um Holter de 24 horas, obtido três meses mais tarde, depois da suspensão da amiodarona, registrou um episódio sincopal – respiração agônica, cianose, movimentos convulsivos – durante o sono no colo de sua mãe. A faixa de ECG apresentou uma ampla variação de ciclo RR, com episódios intermitentes de taquicardia atrial (TA), que se tornou sustentada de condução atrioventricular (AV) de 1:1, com alargamento progressivo do complexo QRS, seguido de taquicardia ventricular (TV) polimórfica, fibrilação ventricular (FV) e 30 segundos de assistolia; o ritmo sinusal foi restaurado espontaneamente (Figura 2).

Houve uma nova ocorrência de taquicardia com QRS alargado na unidade de terapia intensiva. A infusão de adenosina 1 mg nesse momento revelou um novo FLA antes de uma nova cardioversão elétrica.

Estudo eletrofisiológico (EEF) foi realizado sob sedação intravenosa profunda (quetamina 0,2 mg/kg e infusão contínua de propofol) com dois cateteres multipolares 5F. Detectou-se um aumento do intervalo HV (63 ms), e FLA típico, dependente do istmo cavo tricuspídeo (ICT) foi induzido por estimulação atrial programada. Foi realizada ablação linear por cateter de radiofrequência (RF) com bloqueio bidirecional da condução pelo ICT. A estimulação programada (2 ciclos e 2 extra-estímulos) não induziu arritmias ventriculares.

Testes genéticos identificaram uma mutação patogênica heterozigótica do gene SCN5A - variante c.362G>A p. (Arg121Gln) no éxon 3 do gene SCN5A (NM_198056), compatível com a SB.

O paciente é filho único, sem histórico familiar de arritmias, síncope ou MS. A mãe, de 30 anos de idade, apresentou ECG normal e painel genético negativo. O pai, de 34 anos e assintomático, apresentou um bloqueio AV de primeiro grau (PR = 220 ms) e padrão típico de SB Tipo I no ECG (Figura 1), intervalo HV anormal (73 ms), ausência de arritmias ventriculares induzíveis e mesma mutação do gene SCN5A.

Depois de 18 meses de acompanhamento sem sintomas ou EA, apresentou um novo episódio sincopal, desencadeado por febre. Não foram observadas alterações eletrocardiográficas, tanto no ECG de repouso como no Holter de 24 horas. Nesse momento, foi implantado um cardioversor desfibrilador implantável (CDI) de câmara única por via transvenosa. No acompanhamento de 6 meses, não foram observadas complicações relacionadas ao dispositivo, EA ou terapias do CDI.

## Discussão

O presente estudo relatou o caso de um menino de 2 anos com um coração aparentemente normal com EA potencialmente fatal desencadeada por TA sustentada. Esse evento incomum nos levou a suspeitar de uma possível canalopatia. Foi detectada mutação no SCN5A na criança e em seu pai, que apresentou um padrão de SB em ECG Tipo I.

Desde a descrição inicial da doença em 1992, que incluiu três crianças,^3^ os dados sobre a população pediátrica com SB publicados são muito limitados. O padrão de ECG típico da SB (tipo I - elevação côncava do ST nas derivações precordiais direitas) e as manifestações clínicas geralmente não são observadas em crianças mais novas. A idade do aparecimento dos sintomas e EA varia entre 40 e 50 anos, e eles são raros em crianças e idosos.^5^ Na SABRUS (*Survey on Arrhythmic Events in Brugada Syndrome* - Pesquisa sobre Eventos Arrítmicos na Síndrome de Brugada), que incluiu 678 pacientes com SB, a grande maioria (94,2%) dos pacientes tinha entre 16 e 70 anos no momento do primeiro EA, enquanto pacientes pediátricos (<16 anos) e idosos (>70 anos) representavam 4,3% e 1,5%, respectivamente.^8^ A síncope geralmente é a primeira manifestação clínica em 14 a 21%, e a MS acontece em 5 a 7% dos pacientes pediátricos com SB, mas a maioria é assintomática.^9^^,^^10^ A predominância significativa de pacientes do sexo masculino existente entre adultos não é observada em crianças na pré-puberdade, possivelmente devido a influências hormonais, especialmente os níveis de testosterona.^5^^,^^11^

Em nosso paciente, o EA ocorreu durante o sono, como é frequentemente descrito em pacientes com SB, o que sugere uma associação com bradicardia e possivelmente modulação vagal. No presente caso, o evento arrítmico sincopal registrado no Holter de 24 horas – TV/FV – foi desencadeado por uma TA com condução AV rápida durante o sono, sugerindo alguma influência vagal. Sabe-se também que a febre comumente desencadeia arritmias (e pode revelar o padrão típico de ECG),^6^^,^^12^ e ela foi observada no segundo evento sincopal do paciente deste estudo. Há alguns relatos na literatura de arritmias potencialmente fatais e MS em pacientes muito jovens com SB, mas nenhum claramente documentou uma participação direta de uma TA na indução de TV/FV.

A estratificação de risco em pacientes jovens ainda continua a ser um desafio. O padrão de ECG tipo I, síncope, MS, disfunção do nó sinusal, arritmias atriais, anormalidades na condução, e arritmias ventriculares induzidas em EEF foram descritos como preditores de eventos potencialmente fatais.^9^^,^^10^

Nenhum estudo amplo comprovou que a presença de uma mutação do SCN5A é um marcador de risco. Entretanto, mutações complexas do SCN5A parecem levar fenótipos mais graves.^13^

Embora essa criança, desde o evento inicial, tenha atendido aos critérios para CDI, os riscos potenciais de um CDI em crianças muito jovens (terapias inapropriadas e complicações relacionadas aos eletrodos) foram levados em consideração na decisão de adiar o implante. Além disso, a possibilidade da ablação da arritmia desencadeadora de FV (FLA) nos levou a acreditar em uma chance menor de recorrência precoce.

## Conclusão

O caso apresentado demonstra uma apresentação grave de arritmia numa criança, que foi diagnosticada com uma mutação no gene SCN5A. O circuito desencadeador de TV/FV - flutter atrial - foi eliminado por ablação, resultando em alívio dos sintomas por um longo período, mas o implante CDI foi realizado devido à recorrência da síncope. Isso reforça o quão complexa pode ser a apresentação e a evolução de algumas canalopatias na população pediátrica.
