# Supplementary material for: Arritmia Ventricular Potencialmente Fatal Induzida por Taquicardia Atrial em uma Criança com Mutação de SCN5A
Source: Arq Bras Cardiol. 2021 Jul 14;117(1 Supl 1):19–22. [Article in Portuguese] doi: 10.36660/abc.20200509 (PMC8291895; doi:10.36660/abc.20200509)
Supplement: Translation

# Translation: Life-Threatening Ventricular Arrhythmia Induced by Atrial Tachycardia in a Child with an SCN5A Mutation

**Authors:** Márcio Augusto Silva, Jorge Elias, Guilherme Muller de Campos Futuro, Erick Sessa Merçon, Deborah Vasconcelos, Ricardo Kuniyoshi

**Affiliations:** 1 SerraES Brazil Vitoria Apart Hospital - Cardiologia, Serra, ES - Brazil.

**Keywords:** Cardiac Arrhythmia, Brugada Syndrome, Syncope, Child

## Introduction

Mutations in the SCN5A gene, encoding the cardiac Na+channel, can result in several life-threatening arrhythmias. These mutations have proven to be causative for inherited and primarily electrical diseases, including Brugada Syndrome (BrS), Long QT Syndrome, and other cardiac conduction disturbances.^1^^,^^2^ BrS, the most reported condition in this group of disorders, has typically been described in adult populations and related to approximately 20% of all sudden deaths (SD) in patients with apparently normal hearts.^3^^-^^5^ Therefore, few reports have shown significant arrhythmic events (AE) caused by this mutation in childhood.^6^

Atrial arrhythmias, as well as sick sinus syndrome (SSS), may be related to Na+channel abnormalities. In BrS, atrial arrhythmias are being diagnosed in up to 38% of patients and are related to worse prognosis.^7^

## Case Report

A 2-year-old boy, with no cardiac structural anomalies detected in transthoracic echocardiogram and magnetic resonance imaging (MRI) exams, was admitted twice to the hospital with a typical pattern of atrial flutter (AFL), which was reverted by electrical cardioversion. After the last episode, he was discharged on 3 mg/Kg amiodarone daily. The 12-lead ECG showed a normal QT (390 – 410 ms) and QTc (413 – 440 ms) interval, slightly prolonged PR interval (200 ms) and negative T waves in right precordial leads (Figure 1).

**Figure 1:** Spontaneous 12-lead ECG: (A) – Child; (B) – Father.

A 24h-Holter obtained three months later, after amiodarone withdrawal, recorded a syncopal episode – agonal respiration, cyanosis, convulsive movements – while sleeping on his mother's lap. The ECG strap showed a wide RR variation with intermittent atrial tachycardia (AT) that became sustainable with a 1:1 atrioventricular (AV) conduction pattern with a progressive QRS complex enlargement, follow by polymorphic ventricular tachycardia (VT), ventricular fibrillation (VF), and 30 seconds of asystole; the sinus rhythm was spontaneously restored (Figure 2).

**Figure 2:** Syncopal event recorded by 24-Holter. A, B – variable AV conduction AT/AFL, with a narrow QRS; C, D – The AV conduction became 1:1 with progressive QRS enlargement; E, F, G, H – sustained wide polymorphic VT and VF; I, J, K – the VF is followed by 30 seconds of asystole, with spontaneous sinus rhythm recovery; L – long strap of total asystole.

A wide QRS tachycardia recurred in the intensive unit when 1 mg of adenosine IV was administered, unmasking an AFL before a new electrical cardioversion.

An electrophysiological study (EPS) was performed under deep intravenous sedation (ketamine 0.2 mg/kg and continuous propofol infusion) with two 5F multipolar catheters. An increased HV interval (63 ms) was detected and cavo-tricuspid isthmus (CTI) dependent AFL was induced by programmed atrial pacing. Linear radiofrequency (RF) catheter ablation was performed to achieve a bidirectional CTI conduction block. Programmed stimulation (2 cycles and 2 extra-stimulus) did not induce ventricular arrhythmias.

A heterozygous pathogenic SCN5A gene mutation - c.362G>A p. (Arg121Gln) variant in exon 3 of the SCN5A (NM_198056) gene, compatible with BrS, was identified by genetic tests.

The patient is an only child, with no family history of arrhythmias, syncope, or SD. His 30-year old mother had a normal ECG and negative genetic panel. His father, an asymptomatic 34-year old man, showed a first-degree AV block (PR = 220 ms) and typical Type I BrS-pattern on ECG (Figure 1), an abnormal HV interval (73 ms) without inducible ventricular arrhythmias, and the same SCN5A mutation.

After an 18-month follow-up without symptoms or AE, a new syncopal episode occurred, triggered by fever. No new electrocardiographic changes were observed, neither in rest ECG or Holter monitoring. At this time, a transvenous single chamber implantable cardioverter defibrillator (ICD) was implanted. In a 6-month follow-up, no device related complications, AE, or therapies were observed.

## Discussion

The present study described a case of a 2-year old boy with an apparently normal heart with life-threatening AE triggered by a sustained AT. This uncommon event leads us to suspect of a possible channelopathy. SCN5A mutation was detected in the infant and in his father, who presented a Type-I ECG BrS pattern.

Since the initial description of the disease in 1992, which includes three children,^3^ the published data on the BrS pediatric population is very limited. The typical ECG pattern of BrS (type I - ST coved elevation in right precordial leads) and clinical manifestations are not usually seen in young children. The age of onset symptoms and AE range from 40 to 50 years of age is rare in pediatrics or the elderly.^5^ In SABRUS (Survey on Arrhythmic Events in Brugada Syndrome), which includes 678 BrS-patients, the vast majority (94.2%) of the patients were 16-70 years of age at the time of first AE, while pediatric (<16 years) and elderly patients (>70 years) comprised 4.3% and 1.5%, respectively.^8^ Syncope is usually the first clinical manifestation in 14% to 21% and SD in 5% to 7% in pediatric BrS-patients, but the majority are asymptomatic.^9^^,^^10^ The significant male predominance observed in adults is not reported in prepubertal children, possibly due to hormonal influences, particularly testosterone levels.^5^^,^^11^

The AE occurred during sleep in our patient, as is often described in BrS-patients, suggesting an association with bradycardia and possibly vagal modulation. In the present case, the syncopal arrhythmic event registered on a 24h-Holter – VT/VF – was triggered by an AT with a fast AV conduction during sleep, suggesting some vagal influence. Fever is also known to be a common arrhythmic trigger (and may unmask the typical ECG pattern),^6^^,^^12^ and was seen in our patient's second syncopal event. There are a few reports in the literature regarding life-threatening arrhythmias and SD in very young BrS-child patients, but none clearly document a direct participation of an AT on VT/VF induction.

Risk stratification in young patients remains challenging. Type I ECG pattern, syncope, SD, sinus node dysfunction, atrial arrhythmias, conduction abnormalities, and ventricular arrhythmias induced on EPS have been described as predictors of life-threatening events.^9^^,^^10^

The presence of an SCN5A mutation has not been proven to be risk marker in any large study. However, SCN5A compound mutations seem to lead to more severe phenotypes.^13^

Although this child, since the index event, has already met indication criteria for ICD, the potential risks of an ICD in a very young child (inappropriate therapies and lead complications) were taken into account to extend the implant. Moreover, the possibility to ablate the VF-trigger (AFL) led us to believe in a lower chance of early recurrence.

## Conclusion

The presented case demonstrates a severe presentation of an AE in a toddler, who was diagnosed with a SCN5A gene mutation. The VT/VF trigger circuit (AFL) was ablated, resulting in symptom relief over a long period of time, but an ICD was implanted due to syncope recurrence, highlighting how complex a presentation and evolution of some channelopathies can be in a pediatric population.
